# Tunable Broadband THz Waveband Absorbers Based On Graphene for Digital Coding

**DOI:** 10.3390/nano10091844

**Published:** 2020-09-15

**Authors:** Huiping Yang, Dingbo Chen, Yuliang Mao, Junbo Yang

**Affiliations:** 1 Hunan Key Laboratory of Micro-Nano Energy Materials and Devices, School of Physics and Optoelectronics, Xiangtan University, Xiangtan 411105, China; 201821521341@smail.xtu.edu.cn; 2 Center of Material Science, National University of Defense Technology, Changsha 410073, China; chendingbo15@nudt.edu.cn

**Keywords:** tunable, broadband, digital coding

## Abstract

A method of coding patterns is proposed to achieve flexible control of absorption response at terahertz frequencies. The designed absorber consists of an Au-graphene pattern layer, a SiO_2_ layer and a metal reflective layer. Among them, we use concentrical circle structure to achieve broadband absorption, and adjust graphene’s Fermi level to achieve tunable absorption. In addition, we propose an encoding method that can achieve flexible control of the absorption response at the terahertz frequency based on the external voltage applied on the graphene membrane, thereby having a programmable function. We also use COMSOL to simulate the electric field distribution diagram to explain the underlying physical mechanism. The programmable broadband adjustable absorber proposed in this paper has potential application prospects in the fields of optical equipment, information transmission, digital coding and artificial intelligence (AI).

## 1. Introduction

Metamaterials, which are the most influential new materials developed after polymer materials and nanomaterials, are a kind of artificially designed materials that exceed the electromagnetic properties of natural materials. Over the past two decades, due to their designability and feasibility, metamaterials have attracted wide attention from experts in various fields, such as stealth [1,2], radiation protection [3] and communication [4,5]. A particularly striking branch of metamaterials is the “perfect absorber”. In 2008, the Landy research team in the United States achieved perfect absorption of a single frequency and proposed the concept of a perfect absorber [6]. This is a kind of electromagnetic loss caused by the metal plasma structure, so it has attracted people’s attention. Since then, perfect absorbers have developed rapidly, from single band to multi-band [7,8], broadband [9,10,11] and tunable [12,13,14] absorption. Among them, broadband and tunable absorbers have attracted much attention due to their great value in the fields of communication and sensing. Patterns with different resonance frequencies can be coplanar [15] or vertically [16] superimposed to successfully design a broadband absorber. However, in experiments, vertical stacking can make preparation difficult. Tunable absorbers can be successfully designed using materials that can be changed with the external environment, such as ViO_2_ [17] and graphene [18,19]. Nowadays, the digital code absorber may be a new research trend. Digital coding only uses a combination of “0” and “1” to classify different signals. After encoding, the signal can be processed by a computer. In the field of optical transmission, both amplitude and phase are factors to be considered when encoding. The absorber is a structure used to change the amplitude of electromagnetic waves, so it can generate different amplitude information, thereby providing conditions for digital encoding.

In 2014, Professor Cui Tiejun’s research team proposed a digitally encoded metasurface [20]. The binary digits “0” and “1” are used to replace two unit cell structures that can form opposite phases, so that they become digital units. Then using these two types of digital unit cells to construct an all-digital surface. The arrangement of numbers determines the regulation of electromagnetic waves. Digital control facilitates the application of algorithms to the design of metasurfaces. Currently, encoded holograms [21], low-scattering coded metasurface [22] and encrypted encoded absorbers [23] have been reported. Based on the current coding method, if the digital sequence has periodicity, only the nature of the minimum period needs to be simulated and calculated. If the number sequence is irregular, it will increase the amount of calculation during simulation. With this in mind, we think that a special case can be used in absorber: only one unit cell structure is used to construct a surface with identical numbers, where the unit cell structure is the minimum period of the metamaterial absorber. Due to periodicity, the properties of the unit cell represent the properties of the entire absorber, which is easy to calculate. By adding controllable materials to the periodic structure, the structure has adjustable properties. Then, encoding the various tuning states to realize the digital characterization of the absorption spectrum. After digital characterization, the absorber can be combined with a computer program and used to transmit or process spectral signals.

Graphene, the thinnest two-dimensional material found, exhibits metallic properties when it meets particular conditions. Due to its strong interaction with terahertz waves, graphene can be a prospective candidate for perfect absorption at terahertz waves. More importantly, a significant feature of graphene is that the Fermi level can be adjusted by applying a gate voltage. The change of Fermi level will cause the dielectric properties of graphene to be changed, and then achieve the purpose of tuning absorption. Therefore, graphene is a good candidate for tunable absorbers.

In 2018, Meng reported a double-notch metal ring absorber, which achieved a broadband absorption of 3.7 THz [24]. In this paper, we modify the structure proposed by Meng and upgrade the broadband absorber to a tunable broadband absorber by adding graphene to the gaps. The designed structure consists of an Au-graphene pattern layer, a SiO_2_ layer and a metal reflective layer. Based on this structure, we mainly design two absorbers. One is that the pattern layer is an Au-ring with two symmetrical graphene-notches. When the bias voltage external to the graphene is 0 V, the absorption is an ultra-wideband absorption with 3.8 THz whose value is greater than 80% from 1.9 THz to 5.7 THz. When the bias voltage changes, we can achieve adjustable absorption. In addition, we code according to voltage conversion, and realize a programmable 2^2^-element absorber. The other is that the pattern layer is two Au-rings with four graphene-notches. Similar to the first work, when the bias voltage external to the graphene is 0 V, the absorption is an ultra-wideband absorption with 3.74 THz whose value is greater than 89% from 1.95 THz to 5.69 THz. Furthermore, we encode according to voltage changes, and realize a programmable 2^4^-element absorber. Different from phase encoding, our work is to use grapheme’s good controllability to realize the regulation of spectral characteristics, and use this as a method of information encoding.

## 2. Materials and Methods 

**Figure 1:**
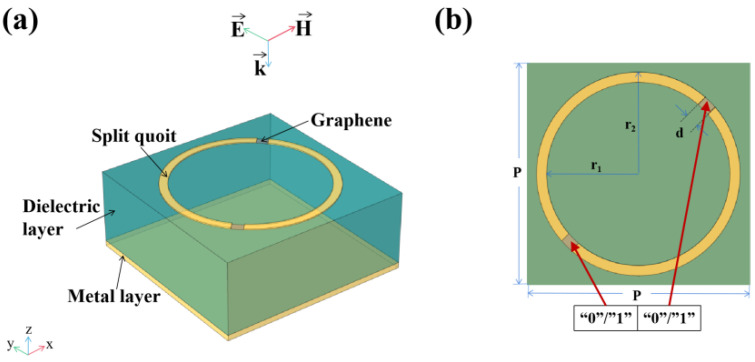
Schematic diagram of double notch single ring metamaterial absorber: (**a**) perspective view of a unit cell; (**b**) top view of a unit cell.

**Figure 2:**
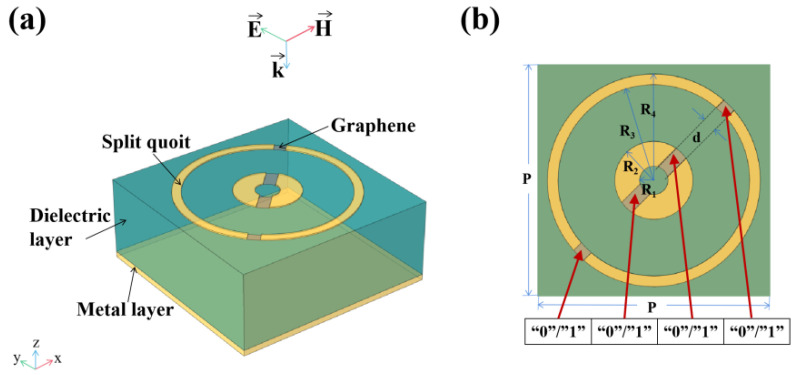
Schematic diagram of four notch dual ring metamaterial absorber: (**a**) perspective view of a unit cell; (**b**) top view of a unit cell.

The metamaterial absorbers we proposed are composed of an Au-graphene pattern layer, a dielectric layer and a metal reflective layer, as shown in Figure 1 and Figure 2 [24]. Figure 1 and Figure 2 show schematic diagrams of a double notch single ring and a four notch dual concentric ring metamaterial absorber, respectively. It can be seen that the notch ring and metal layer are made of lossy gold with a conductivity of $\delta =4.56{\times 10}^{7}\;S/m$, and their thickness is 0.2 μm and 0.4 μm, respectively. As the dielectric layer, SiO_2_ having a dielectric constant of ε=2.13 and a thickness of 13.3 μm is used. Table 1 shows some geometric parameters of the unit cell. It is not negligible that we put graphene in the notches of the notch ring. The way of applying voltage to graphene [25] can be seen in Figures S1 and S2 in Supplementary Materials.

The conductivity of graphene is provided by Kubo’s equation, which is determined by both intra-band and inter-band transition [26]:

(1) $$\sigma_{g}=\sigma_{intra}+\sigma_{inter}$$

(2) $$\sigma_{intra}=\frac{{2k}_{B}{\mathrm{Te}}^{2}}{\pi \hbar^{2}}\times \ln(2\cos\;h\frac{E_{f}}{{2k}_{B}T})\times \frac{i}{\omega +{i\tau }^{-1}}$$

(3) $$\sigma_{inter}=\frac{e^{2}}{4\hbar }\times [H\left(\frac{\omega }{2}\right)+i\times \frac{4\omega }{\pi }\times \int_{0}^{\infty }\frac{H\left(\Omega \right)-H(\frac{\omega }{2})}{\omega^{2}-{4\Omega }^{2}}d\Omega ]$$

here, $H\left(\Omega \right)=\sin\;h\left(\frac{\hbar \Omega }{k_{B}T}\right)/\left[\cos\left(\frac{\hbar \Omega }{k_{B}T}\right)+\cos h\left(\frac{E_{f}}{k_{B}T}\right)\right]$, $k_{B}$ is the Boltzmann’ s constant, T is the temperature, e is the elementary charge, ℏ is the reduced Planck’ s constant, ω is the angular frequency, $\tau =\mu E_{f}/{ev}_{f}^{2}$ is the carrier relaxation time that is a function of carrier mobility ${\mu =10}^{4}\;{\mathrm{cm}}^{2}/V$, the Fermi energy of graphene $E_{f}$ and Fermi velocity $v_{f}=10^{6}\;m/s$.

In the terahertz band, the conductivity of graphene is mainly provided by the intra-band transport of carriers, while the inter-band transport is restrained. At this time, the calculation equation of the conductivity of graphene is simplified:

(4) $$\sigma_{g}\left(\omega \right)=\frac{e^{2}E_{f}}{\pi \hbar^{2}}\times \frac{i}{\omega +{i\tau }^{-1}}$$

The dielectric constant of graphene is expressed as:

(5) $$\varepsilon_{g}\left(\omega \right)=1+\frac{i\sigma_{g}\left(\omega \right)}{\varepsilon_{0}\omega \Delta }$$

Here, $\varepsilon_{0}$ is the dielectric constant of the vacuum, Δ=0.5 nm is the thickness of graphene. Since the wavelength of the electromagnetic wave is much larger than the thickness of graphene, a transition boundary condition can be used in the simulation. This simplified method can reduce the calculation time.

The Fermi level of graphene is determined by the carrier concentration and is given by the Equation (6):

(6) $$E_{f}=\hbar v_{f}\times \sqrt{\pi n_{s}}$$

Here, $n_{s}$ is the carrier concentration. The carrier concentration can be changed by bias voltage and chemical doping. Equation (7) gives the conversion relationship between carrier concentration and applied voltage.

(7) $$n_{s}=a_{0}\times \left|V_{biased}\right|$$

**Figure 3:**
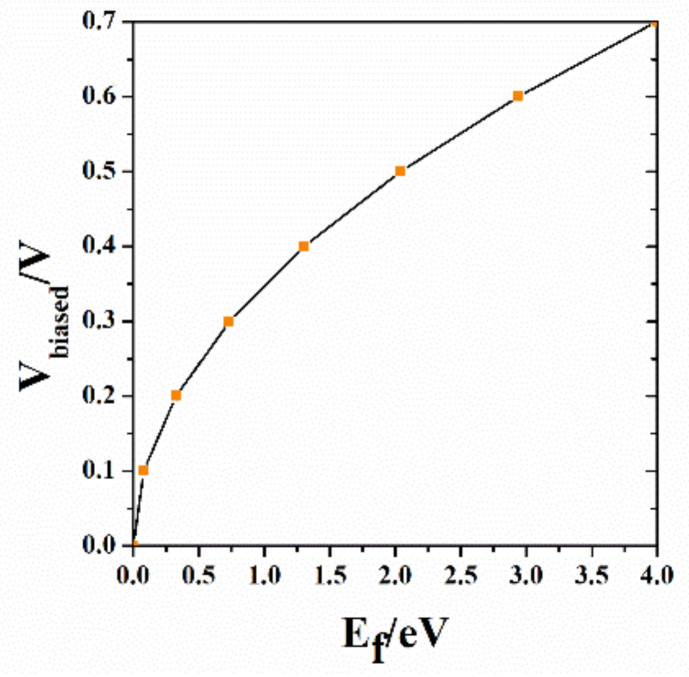
The relationship between the Fermi level of graphene and the bias voltage.

Here, $a_{0}=\frac{\varepsilon \varepsilon_{0}}{eH}$, ε is the dielectric constant of the dielectric layer, H is the thickness of the dielectric layer, $\varepsilon_{0}$ is the dielectric constant of vacuum, $V_{biased}$ is the bias voltage. We calculated the relationship between Fermi level and bias voltage by Equations (6) and (7), as shown in the Figure 3.

It can also be seen from Figure 3 that when the bias voltage is 0 V, the Fermi level is 0 eV. The higher the bias voltage, the larger the Fermi level. When the fermi level is 0 eV, graphene has a relatively small effect on absorption, which is simplified to the absence of graphene in the simulation.

Performance simulations of the metamaterial absorbers are demonstrated using the full-wave electromagnetic simulation software COMSOL Multiphysics 5.4 which based on finite element method. In the simulation, a unit cell in the periodic structure is used as the calculation object, and the *x* and *y* directions are set as the Floquet periodic boundary, and the *z* direction is set as the perfect matching layer (PML). With normal incidence, the reflectance R(ω) and transmittance T(ω) of the absorber can be calculated by COMSOL. Since the thickness of the metal layer is greater than the skin depth through which the electromagnetic waves travel, the transmission is blocked. Therefore, the absorption rate can be expressed as A(ω)=1−R(ω).

## 3. Results and Discussions

### 3.1. Double Notch Single Ring Metamaterial Absorber

**Figure 4:**
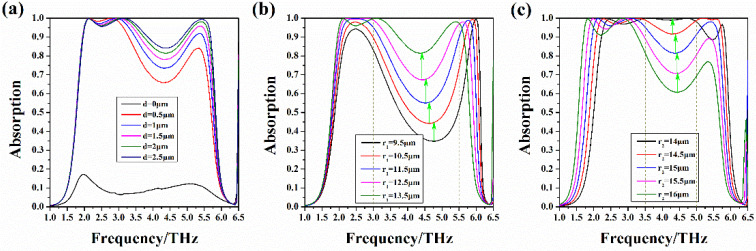
Absorption spectrum of double notch single ring absorbers with $E_{f}=0\;\mathrm{eV}$: (**a**) Absorption spectrum of the absorber as a function of notch width when $r_{1}=13.5\;\mu m$ and $r_{2}=15\;\mu m$; (**b**) Absorption spectrum of the absorber as a function of $r_{1}$ when d=2 μm and $r_{2}=15\;\mu m$; (**c**) Absorption spectrum of the absorber as a function of $r_{2}$ when d=2 μm and $r_{1}=13.5\;\mu m$.

We first calculate the absorption performance of the double notch single ring absorber. When the Fermi level of graphene is 0 eV, the simulated absorption curves of metamaterial absorbers with different parameters are shown in Figure 4. We set the inner diameter $r_{1}$ of the ring to 13.5 μm and the outer diameter $r_{2}$ to 15 μm. As can be seen from Figure 4a, there are obvious resonance peaks in the absorption spectrum. As the notch width d increases, the absorption of the resonance peaks is increased and the absorption bandwidth becomes wider. Then, we take d equal to 2 μm, keep $r_{2}$ unchanged, and change $r_{1}$. Obviously, in the frequency range of 3 THz to 5.5 THz, as $r_{1}$ decreases, the absorption valley becomes higher and the absorption line becomes flatter, as shown in Figure 4b. Figure 4c shows the absorption spectrum obtained by fixing d and $r_{1}$ and adjusting $r_{2}$. We can intuitively see that as $r_{2}$ decreases, in the frequency range of 3.5 THz to 5.5 THz, the bottom of the absorption line moves upward, and the absorption becomes higher.

**Figure 5:**
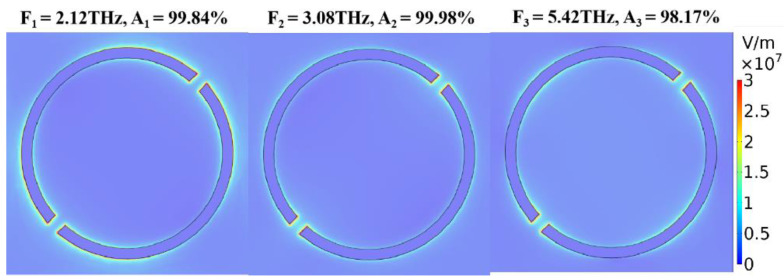
Electric field diagram of three absorption peaks of the single ring, when $E_{f}=0\;\mathrm{eV}$, d=2 μm, $r_{1}=13.5\;\mu m$ and $r_{2}=15\;\mu m$.

In the case of $E_{f}=0\;\mathrm{eV}$, when d=2 μm, $r_{1}=13.5\;\mu m$ and $r_{2}=15\;\mu m$, the absorption is an ultra-wideband absorption with 3.8 THz whose value is greater than 80% from 1.9 THz to 5.7 THz. Figure 5 is an electric field diagram of three absorption peaks under this structure. It can be seen that the three resonance modes correspond to three absorption peaks, and the overlap of the absorption peaks constitutes a broadband absorption. For the following calculations, the structural parameter we use are d=2 μm, $r_{1}=13.5\;\mu m$ and $r_{2}=15\;\mu m$.

We introduce two coding numbers “0” and “1”, which correspond to the two states of graphene controlled by zero voltage and non-zero voltage, respectively, and independently control the switching states at the two gaps in the unit cell. According to the definition above, the patterns are sequenced with a set of binary coding, where the first number corresponds to the gap in the lower left corner and the second number corresponds to the gap in the upper right corner, encoding in the order indicated by the red clips in the Figure 1b. Since there are two options of “0” and “1” at each gap, 2^2^ patterns can be obtained by this coding method. Because the unit cells are symmetrical about the diagonal line, the patterns corresponding to the two coding sequences “01” and “10” have the same geometric information.

**Figure 6:**
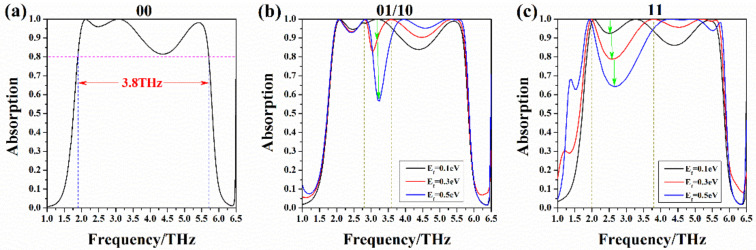
Function of absorption spectrum and Fermi levels for different coding patterns of single ring, where d=2 μm, $r_{1}=13.5\;\mu m$, $r_{2}=15\;\mu m$: (**a**) “00”: Absorption spectrum of two graphene with zero voltage applied; (**b**) “01/10”: When the external voltage of one graphene is 0 V and the external voltage of the other graphene is not 0 V, the relationship between the absorption line and the Fermi level. The Fermi level of non-zero voltage graphene changed from 0.1 eV to 0.5 eV; (**c**) “11”: When both graphenes are applied with the same non-zero voltage, the relationship between the absorption line and the Fermi level. The Fermi level changed from 0.1 eV to 0.5 eV.

After coding the patterns, we calculate the relationship between the absorption spectrum and Fermi level under different patterns, as shown in Figure 6. We discuss the case where zero voltage is applied to two graphenes with gaps “00”, one of graphene is applied with zero voltage “01/10”, and the case where both graphenes are applied with the same non-zero voltage “11”. Firstly, Figure 6a shows the absorption spectrum coded as “00”. This is broadband absorption. The bandwidth with an absorption rate exceeding 80% is 3.8 THz, from 1.9 THz to 5.7 THz. Secondly, Figure 6b shows the absorption spectrum coded as “01” and “10”. It can be seen from the figure that when we adjust the Fermi level of non-zero voltage graphene from 0.1 eV to 0.5 eV, the absorption line corresponding to the frequency range of 2.8 THz to 3.6 THz moves downward. Thirdly, Figure 6c shows the absorption spectrum coded as “11”. We use the same non-zero voltage to control two pieces of graphene. When the Fermi level of graphene changes from 0.1 eV to 0.5 eV, the corresponding absorption line in the frequency range of 2 THz to 3.8 THz shifts down significantly. It can be found that the absorptivity of the four coding patterns show different changing rules.

**Figure 7:**
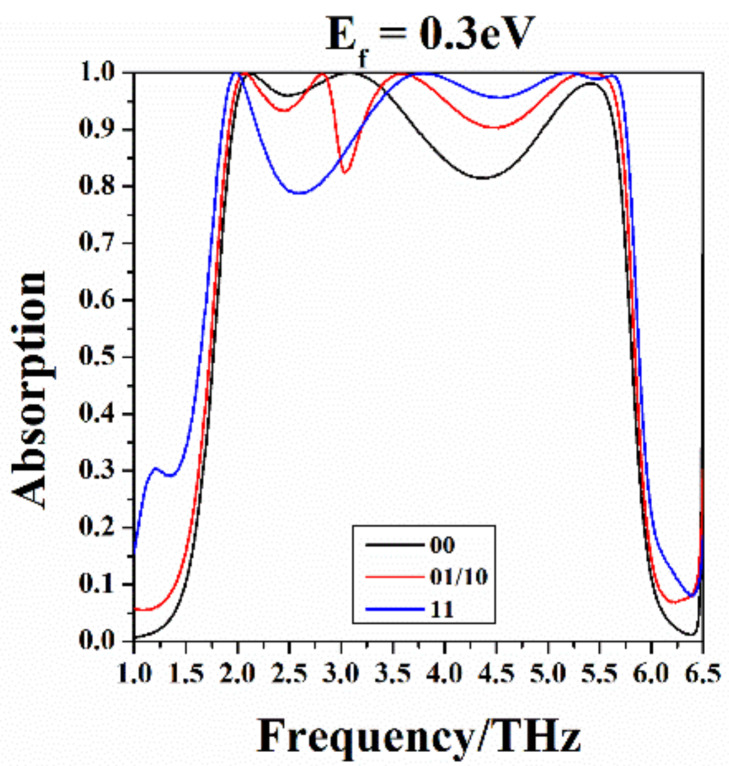
The absorption curves of the four coding patterns. Here, the graphenes marked with “0” have a Fermi level of $E_{f}=0\;\mathrm{eV}$, and the graphenes labeled “1” have a Fermi level of $E_{f}=0.3\;\mathrm{eV}$.

Afterwards, we set the Fermi level of graphene to which non-zero voltage is applied to 0.3 eV uniformly, and compare the four coding patterns’ absorption curves in Figure 7. As can be seen, with different coding patterns, even if the Fermi level of graphene under voltage is the same, their absorption spectra are significantly different. Therefore, it is easy to distinguish the codes by spectral lines.

**Figure 8:**
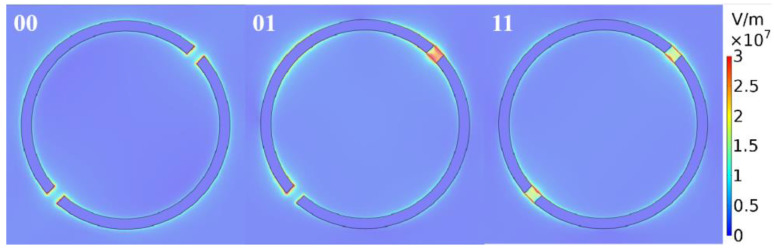
The field intensity distribution of the reflected light on the Au-graphene single ring at the highest absorption efficiency. Here, the graphenes labeled “0” have a Fermi level of $E_{f}=0\;\mathrm{eV}$, and the graphenes labeled “1” have a Fermi level of $E_{f}=0.3\;\mathrm{eV}$.

Each code has its own maximum absorption efficiency. Therefore, we can distinguish coding patterns based on the maximum absorption. Table 2 gives the highest absorption efficiency corresponding to the code and its resonance frequency. Then, we draw the field intensity distribution of the reflected light on the Au-graphene single ring, as shown in Figure 8. Obviously, each coded absorption resonance occurs at a different position. Therefore, we can derive the absorption by coding, and we can also determine the coding by absorption. The interconnection of coding and absorption is established.

Adjusting the Fermi level of graphene not only affects the absorption rate, but also changes the absorption bandwidth. Compared with the narrowband absorber, the bandwidth of the broadband absorber has a larger fluctuation range. This helps us to distinguish tuning codes based on the difference in absorption bandwidth. In order to reflect the relationship between the absorption bandwidth and the encoding, we calculate the absorption bandwidth of the encoded absorption spectrum appearing in Figure 7, as shown in Table 3. It can be seen that when the absorption is greater than 90%, the bandwidth of each code is different. Therefore, we can identify each code based on the absorption bandwidth.

### 3.2. Four Notch Dual Ring Metamaterial Absorber

In addition, we calculate the absorption performance of the four-notch double ring absorber. Similarly, we introduce two coding numbers “0” and “1”, which correspond to the two states of graphene controlled by zero voltage and non-zero voltage, respectively, and control the switching states at four gaps in unit cells. According to the definition above, the pattern is sequenced by a set of four digits. The first digit to the last digit of the array, respectively, correspond to the voltage application of the four gaps in the order from bottom left to top right, as shown by the red arrow in Figure 2b.

**Figure 9:**
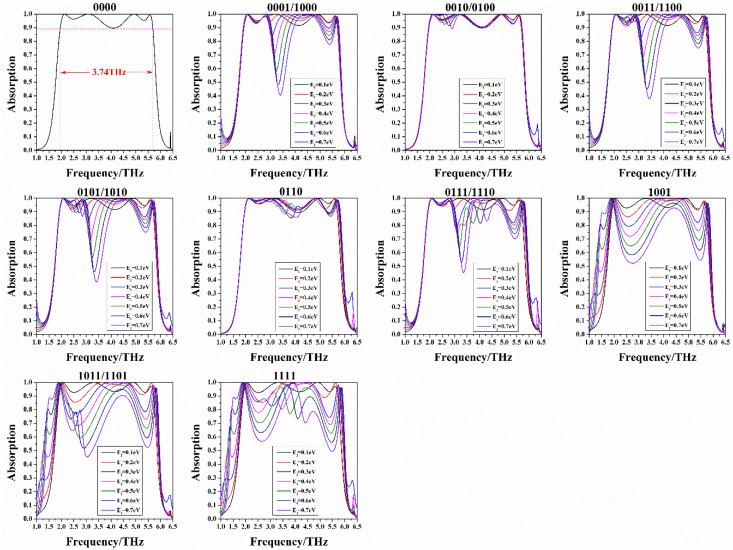
Function of absorption spectrum and Fermi levels for different coding patterns of dual ring. 0000, 0001, 0010, 0011, 0100, 0101, 0110, 0111, 1000, 1001, 1010, 1011, 1100, 1101, 1110 and 1111 are 16 encoding methods. Among them, there are six pairs of codes with symmetry. The sub-picture is the tuning spectrum corresponding to various codes.

Since there are two options of “0” and “1” at each gap, 2^4^ patterns can be obtained by this encoding method. Because the unit cell is symmetrical about the diagonal, there are six sets of coding patterns with the same geometric information, in fact there are only 10 different patterns. After coding the modes, we calculate the absorption spectra of these 16 codes, as shown in Figure 9. The code “0000” is the case where the Fermi level of all graphenes is 0 eV. In the case of, the absorption is ultra-wideband absorption with 3.74 THz whose value is greater than 89% from 1.95 THz to 5.69 THz. For the other fifteen types of codes with the Fermi level that is not all 0 eV, we change the non-zero Fermi level from 0.1 eV to 0.7 eV. As the Fermi level is adjusted, its absorption spectrum changes significantly. Tunability is thus achieved by changing a voltage.

**Figure 10:**
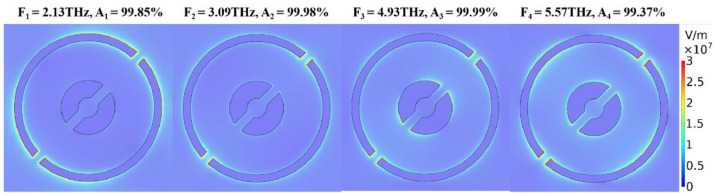
Electric field diagram of four absorption peaks of the double ring, when zero voltage is applied to the four graphenes.

In order to explain the absorption mechanism of the device, the electric field diagrams of the four absorption peaks under the “0000” structure were simulated, as shown in Figure 10. It can be seen that the four resonance modes correspond to four absorption peaks, and the overlap of the absorption peaks constitutes a broadband absorption.

**Figure 11:**
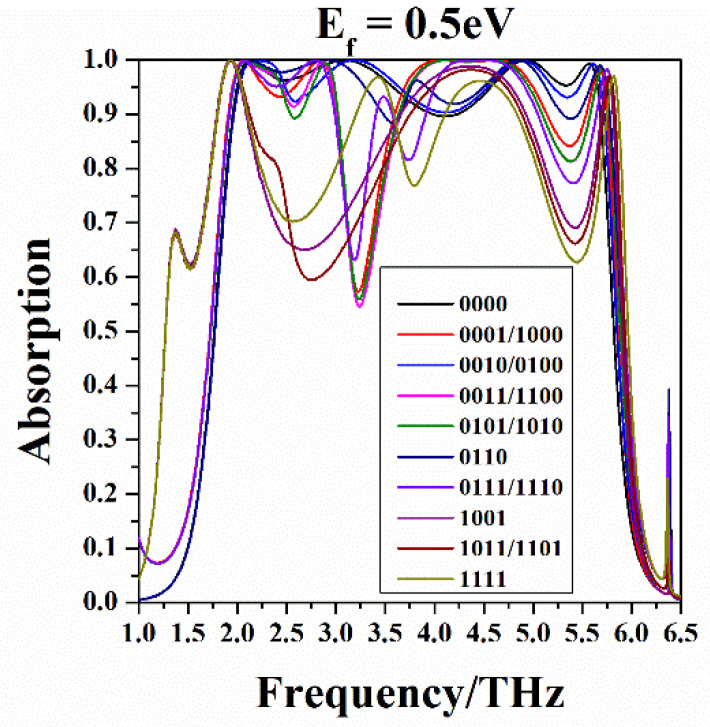
The absorption curves of the sixteen coding patterns, here, the graphenes marked with “0” have a Fermi level of $E_{f}=0\;\mathrm{eV}$, and the graphenes labeled “1” have a Fermi level of $E_{f}=0.5\;\mathrm{eV}$.

Following, the graphenes labeled “0” are controlled by zero voltage and the Fermi level of graphenes labeled “1” are uniformly adjusted to 0.5 eV, drawing the absorption spectrum in the Figure 11. Comparing the 16 codes’ absorption curves in Figure 11, it can be seen the absorption spectrum can be obtained by coding or the code can be identified by the absorption spectrum, which means that these codes can be distinguished.

**Figure 12:**
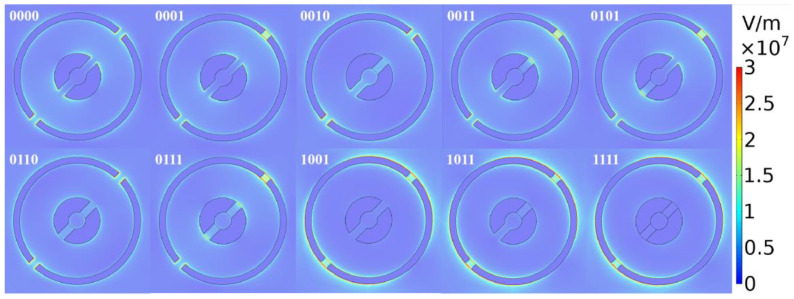
The field intensity distribution of the reflected light on the Au-graphene dual ring at the highest absorption efficiency. Here, the graphenes marked with “0” have a Fermi level of $E_{f}=0\;\mathrm{eV}$, and the graphenes labeled “1” have a Fermi level of $E_{f}=0.5\;\mathrm{eV}$.

Table 4 is the data of the highest absorption efficiency and its resonance frequency corresponding to each code. In Figure 12, we plot the field intensity distribution of the reflected light on the Au-graphene single ring. The results show that the absorption resonance of each code is caused by resonance at different positions.

In addition to the method of distinguishing codes introduced above, we can also distinguish codes based on absorption bandwidth. We calculate the absorption bandwidth of the coded absorption spectrum appearing in Figure 11. In Table 5, we compare the bandwidth of each code (absorption is greater than 90%) and find that the absorption bandwidth of each code is different, so the method of judging the code by the absorption bandwidth is also feasible.

It can be seen that each tuning code corresponds to a different absorption spectrum (different absorption and different bandwidth). Therefore, the transmission and exchange of information can be realized by using electromagnetic waves as the carrier and digital coding and spectrum as the conversion interface.

## 4. Conclusions

In conclusion, we propose an encoding method to achieve flexible control of the absorption response at terahertz frequencies. For the terahertz band, an Au-graphene double notch single ring absorber is designed. Utilizing the principle of overlapping absorption peaks, ultra-wideband absorption of 3.8 THz can be achieved when the Fermi energy level of graphene is all 0 eV, and the absorption rate exceeds 80%. The Fermi level of graphene is then adjusted from 0.1 eV to 0.5 eV to achieve adjustable absorption. More importantly, we use the voltage of the two graphenes in the structure to encode, thus achieving a programmable 2^2^-element absorber. After that, we add a gap ring. When the Fermi energy level of graphene is all 0 eV, ultra-wideband absorption of 3.74 THz is achieved, and the absorption rate exceeds 89%. The Fermi level of graphene is then adjusted from 0.1 eV to 0.7 eV to achieve adjustable absorption. The voltage applied to graphene is then used to encode at four locations in the structure, and a 2^4^-element programmable absorber is implemented, which expand the encoding range. Our design is not only useful for broadband transmission and tuning signals, but also has programming capabilities. It is a combination of electromagnetic artificial materials and information science, which has important reference value for promoting intelligent devices and intelligent operations.

## Figures and Tables

**Table 1:** Some geometric parameters of the unit cell.

Parameter	*P*/μm	*r*_1_/μm	*r*_2_/μm	*R*_1_/μm	*R*_2_/μm	*R*_3_/μm	*R*_4_/μm	*d*/μm
Value	35.0	13.5	15.0	2.0	5.5	13.5	15.0	2.0

**Table 2:** Maximum absorption efficiency and resonance frequency corresponding to the codes.

Code	00	01	11
Resonance frequency/THz	3.08	5.43	5.20
Absorption efficiency	99.98%	99.97%	99.99%

**Table 3:** Bandwidth of each code (absorption is greater than or equal to 90%).

Code	00	01	11
Bandwidth/THz	2.46	3.51	2.91

**Table 4:** Maximum absorption efficiency and resonance frequency corresponding to the codes.

Code	0000	0001	0010	0011	0101	0110	0111	1001	1011	1111
Resonance frequency/THz	4.93	4.62	3.19	4.56	4.57	2.91	4.46	1.93	1.94	1.93
Absorption efficiency	99.99%	99.99%	99.99%	99.99%	99.99%	99.99%	99.98%	99.97%	99.96%	99.96%

**Table 5:** Bandwidth of each code (absorption is greater than or equal to 90%).

Code	0000	0001	0010	0011	0101	0110	0111	1001	1011	1111
Bandwidth/THz	3.47	2.82	3.74	2.70	2.64	3.45	2.52	1.66	1.55	1.49

## Supplementary Materials

The following are available online at https://www.mdpi.com/2079-4991/10/9/1844/s1, Figure S1: The structure diagram of the applied voltage, Figure S2: Comparison of absorption spectrum with or without ion gel.
