# Supplementary material for: Tunable Broadband THz Waveband Absorbers Based On Graphene for Digital Coding
Source: Nanomaterials (Basel). 2020 Sep 15;10(9):1844. doi: 10.3390/nano10091844 (PMC7558160; doi:10.3390/nano10091844)
Supplement: Supplementary file 1 [file nanomaterials-10-01844-s001.pdf]

nanomaterials

Supplementary Materials

Tunable Broadband THz Waveband Absorbers Based On Graphene for Digital Coding

Huiping Yang [1], Dingbo Chen [2], Yuliang Mao [1,*] and Junbo Yang [2,*]

[1] Hunan Key Laboratory of Micro-Nano Energy Materials and Devices, School of Physics and Optoelectronics, Xiangtan University, Xiangtan 411105, China
[2] Center of Material Science, National University of Defense Technology, Changsha 410073, China
* Correspondence: ylmao@xtu.edu.cn (Y.M.); yangjunbo@nudt.edu.cn (J.Y.)

The way to apply voltage to graphene

Considering that the concept we put forward needs to be combined with the actual door control structure, taking the single-ring absorber as an example, we draw the structure diagram of the applied voltage. As shown in Figure S1, a conductive film electrode is added between the pattern layer and the SiO_2 layer, an ion gel pattern is coated on the graphene, and the Au electrode is connected to the ion gel, so as to realize the voltage adjustment of the Fermi level.

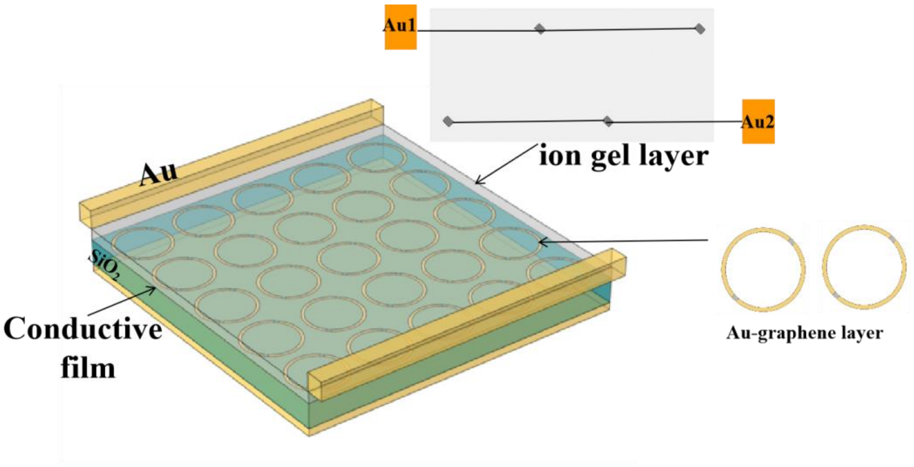

Figure S1. The structure diagram of the applied voltage.

To clarify the effect of ion gel on absorption, we simulated the absorption spectrum of models with or without ion gel. The Fermi levels of the two graphenes in the single-ring absorber are 0.5 eV. The dielectric constant and thickness of the ion gel are 1.82 and 200 nm, respectively. The absorption spectrum is shown in Figure S2. It can be seen that the presence or absence of ionic gel in the simulation has little effect on the absorption.

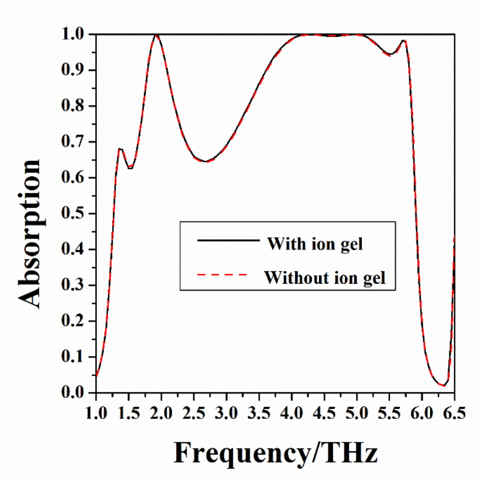

Figure S2. Comparison of absorption spectrum with or without ion gel.
